# The opportunities of semiconductor fibres in clinical and translational medicine

**DOI:** 10.1002/ctm2.1685

**Published:** 2024-06-14

**Authors:** Zhixun Wang, Qichong Zhang, Ming Chen, Lei Wei

**Affiliations:** ^1^ School of Electrical and Electronic Engineering Nanyang Technological University Singapore Singapore; ^2^ Key Laboratory of Multifunctional Nanomaterials and Smart Systems Suzhou Institute of Nano‐Tech and Nano‐Bionics Chinese Academy of Sciences Suzhou China; ^3^ Center for Photonics Information and Energy Materials Shenzhen Institute of Advanced Technology Chinese Academy of Sciences Shenzhen China

**Keywords:** catheter, healthcare wearable devices, semiconductor fibre

## 1 INTRODUCTION

**FIGURE 1:**
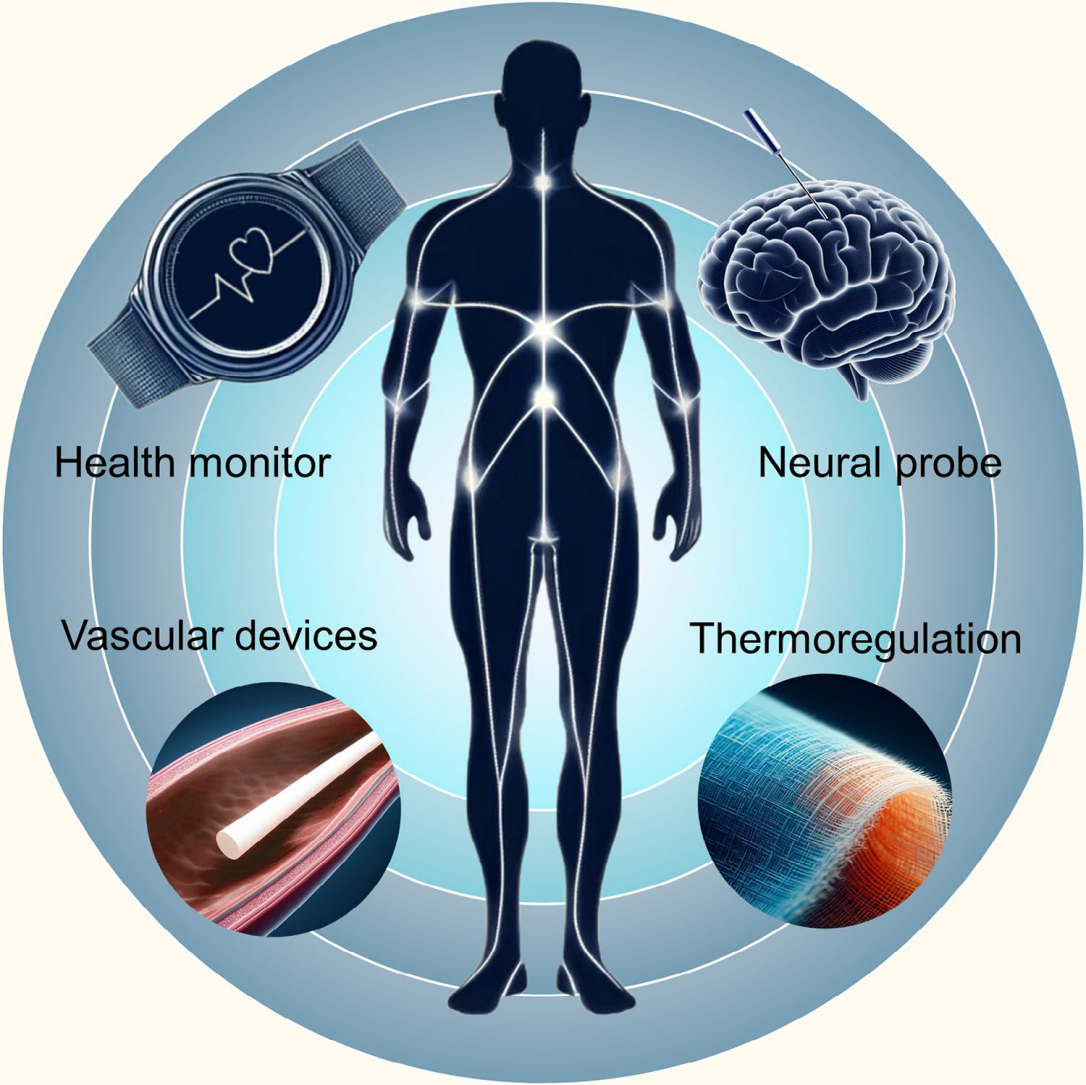
FIGURE 1 Potential applications of semiconductor fibres in clinical and translational medicine.

Measurements, monitoring, and tuning of body processes such as muscle contraction, thermoregulation, circulation and digestion hold a significant place in the field of clinical and translational medicine. From pre‐treatment evaluation and treatment effectiveness tracking to managing chronic diseases, crucial diagnostic information is provided from those measurements and monitoring via medical devices and systems.^1^, ^2^ Creative translational medical devices and systems play a vital role in advanced health care. From the materials perspective, semiconductors are an essential type for those advanced devices that come into play. For example, semiconductors are used in sensors, and imaging systems to monitor various physiological parameters. From the form factor perspective, fibre‐shaped devices have long been used in medicine. Equipped with micro‐optical fibres, the endoscope is advanced in providing high‐resolution imaging and enhanced diagnostic capabilities. Catheters, usually made of polymeric fibres, are used in medicine for various purposes, including drainage, and administration of fluids and medications. Thus bringing the semiconductor materials in the fiber form factor closer together may reach the “best of both worlds” scenario. Conventional semiconductor materials such as silicon (Si) and germanium (Ge) are brittle inorganic crystalline materials, challenging their fabrication in fibre form. Our latest study unveiled that the thermally‐drawn fibre technique can fabricate semiconductor fibre in high quality, high yield and extended single‐strand length through thermomechanical optimization.^3^ From submicrons to hundreds of microns, the controllable thickness of these semiconductor fibres makes them suitable for use and integration into different kinds of medical devices. There's still much to learn on establishing and optimizing their connectivity to medical devices, semiconductor fibres, not expected to replace their planar‐type counterparts, will certainly unlock new and exciting opportunities in clinical and translational medicine (Figure 1).

## 2 THERMOREGULATION

Thermoelectric semiconductors convert heat into electricity (Seebeck effect) or vice versa (Peltier effect). This feature brings them applications in power generation, temperature sensing, and regulation. Our recent work delivered tin selenide fibres.^4^ In the fibre form factor, the thermoelectric semiconductor possesses remarkable characteristics such as being lightweight, thin, and mechanically flexible.^4^, ^5^, ^6^ Breathable functional fabric that offers long‐term comfort can be prepared using these tin selenide fibres. The functional fabric measures body temperature and outputs electrical signals. A woven fibre crossbar structure offers multiple sensing nodes. Each node is created on the crossing of two fibres. 2N number of fibre can form N^2^ nodes, and thus, the functional fabric allows the monitoring of a large area covering the body. This functional fabric provides thermoregulation through the other working modes of thermoelectric semiconductors. Power input generates two sides, the hot and cold sides and polarity switching ensures that the side with skin contact is adjusted on demand.

## 3 PULSE MONITORING

Photoplethysmography (PPG) is a technique that can measure heart rate, mainly using a light source and a photodetector. A small portion of the incident light is scattered back to the photodetector and generates an electrical signal. As the volume of blood vessels changes with the heartbeats, the electrical signal from the photodetector is modulated by the heart rate. A flat and nonconformal photodetector is usually installed for conventional fingertip PPG devices or smartwatches. The measurements are conducted with the testee remaining still to ensure decent contact between the photodetector and skin, which challenges continuous monitoring of such a physiological parameter. In our study, we employed semiconductor fibres to prepare a fibre photodetector, which is soft and flexible to be woven into a wrist strap. This functional strap is conformal to the wrist and is able to measure pulse through PPG. Due to its flexible nature, this sensor provides more accurate monitoring when the testee is active and is suitable for 24‐h continuous monitoring.

## 4 FUTURE PERSPECTIVES FOR SEMICONDUCTOR FIBERS IN CLINICAL AND TRANSLATIONAL MEDICINE

The study suggests that semiconductor fibres have diverse clinical and translational medicine capabilities. The potential of semiconductor materials in such a fibre form factor lies in various applications such as implantable sensors, neural interfaces and tissue engineering. The form factor of semiconductor fibres promotes their integration in catheters and other devices for insertion. As one of many examples, a waveguide of extended wavelength can be integrated into an endoscope using semiconductor fibres. Also, they can be used as neural probes for interfacing with the nervous system. As part of the invasive neural probe, semiconductor fibres can be used to record neural signals, stimulate neural activity either by electrical or optical signals and establish bidirectional communication between the brain or spinal cord and external terminals. Furthermore, biocompatible and bioresorbable semiconductor fibres can be utilized as scaffolds for tissue engineering and regenerative medicine. They might be employed as mechanical support and through stimuli or medicine delivery to promote tissue repair and regeneration. Nevertheless, more understanding of the biophysics of semiconductor fibres will be needed for practical evaluation of their use in clinical usefulness. We hope scientific breakthroughs and advanced technologies continue to emerge and improve in the field to unlock the clinical roles and potential therapeutic benefits for semiconductor fibres.

## AUTHOR CONTRIBUTIONS

All authors have contributed to writing the manuscript and have approved the final manuscript.

## CONFLICT OF INTEREST STATEMENT

The authors declare no conflicts of interest.

## ETHICS STATEMENT

Not applicable.
